# New species of springtails in the *Proisotoma* genus complex from Vermont and New York, USA with descriptive notes on *Ballistura alpa* Christiansen & Bellinger 1980 (Hexapoda, Collembola, Isotomidae).

**DOI:** 10.3897/zookeys.147.2093

**Published:** 2011-11-16

**Authors:** Felipe N. Soto-Adames, Rosanna Giordano

**Affiliations:** Illinois Natural History Survey, University of Illinois, Champaign, IL 61820, USA

**Keywords:** Freshwater lake sandy beach, Lake Champlain, Lake Willoughby, Great Averrill Pond, Chaetotaxy, *Ballistura*, *Pachyotoma*, *Scutisotoma*, *Subisotoma*

## Abstract

Three new species of Isotomidae springtails are described from the Lake Champlain Basin (Vermont and New York, USA), Lake Willoughby and Greater Averril Pond in Vermont. *Subisotoma joycei* **sp. n.** and *Scutisotoma champi* **sp. n.** were collected in sandy beaches whereas *Ballistura rossi* **sp. n.** was found only in a constructed wetland built and managed by the University of Vermont. *Scutisotoma champi* **sp. n.** was found in Lakes Champlain and Willoughby, and Greater Averril Pond and is probably present in most lakes and large ponds in the area. *Subisotoma joycei* **sp. n.** was found only along the southern and eastern coast of South Hero, and the mainland coast facing eastern South Hero. *Ballistura alpa* is redescribed and transferred to the genus *Pachyotoma* based on the absence of tibiotarsal seta B4/B5, the presence of secondary cuticular granules, 4 prelabral setae, a full complement of guard setae on labial papilla E and in having a bifurcate outer maxillary lobe with 4 sublobal setae.

## Introduction

The springtail fauna of Vermont is poorly known. The most comprehensive list includes only 57 species (Bellinger 1982). Most of the springtail collections studied by Bellinger (1982) were made by Ross and Joyce Bell, and their students at the University of Vermont in the highlands around Camel’s Hump. To date the fauna of the Vermont side of the Lake Champlain basin remains mostly unexplored.

As part of a general survey of the springtail fauna of sandy beaches on the Vermont side of Lake Champlain four species in the family Isotomidae belonging to the *Proisotoma* genus complex were collected. Further analysis showed that the samples included *Proisotoma minuta* (Tullberg), 1871 and three undescribed species. In the present contribution we describe the new species and provide additions to the description of *Ballistura alpa* Christiansen & Bellinger, 1980.

## Methods

Most of the terminology used in the descriptions follows Potapov (2001), but we retain the use of dorsal and ventral to identify the faces of the furcula (posterior and anterior, respectively, in Potapov 2001). Postlabial chaetotaxy (other than the number of setae along the ventral groove) has not been used to diagnose species in this group of Isotomidae and its utility is uncertain. However, the general organization of the chaetotaxy in the forms described here is the same as in Lepidocyrtini entomobrids (Soto-Adames 2010), suggesting it may be useful to distinguish species among members of the *Proisotoma* genera complex. The postlabial chaetotaxy (Fig. 3) is described following the nomenclature and column delimitation conventions of Soto-Adames (2010). The columns of setae are designated as (I)nner, (C)entral, (E)xternal, and (L)ateral. The level of intraspecific variation in the number of setae in each column differs among species. The 5 individuals of *Subisotoma joycei* sp. n. studied showed variation in the number of setae in all postlabial columns, but no variation in number of setae was observed in 19 individuals of *Scutisotoma champi* sp. n. Columns I and E are most stable. Column C is usually not well organized and is probably best described as a field of setae; this column also shows the largest amount of intraspecific variation in the species examined (cf Figs 20–22).

The number of postlabial setae and tergal sensilla are often variable and the formula is presented in the format x (y) where x is the mode and y represents other setae number observed. If there is more than one mode the formula is expressed as x/z (y). Separation between thoracic and abdominal tagma is represented by //, segments within tagma are separated by a semicolon (;). Number of setae are given for half a tergite (i.e., formula 10//101 should be understood as 10//101 + 10//101)

Abbreviations used in the descriptions that follow are: Ant., Th., Abd., L and PAO for antenna, thorax, abdomen, leg and postantennal organ.

The types of the new species are deposited in the Insect Collection of the Natural History Survey, at the University of Illinois, Champaign, IL, USA. The slides of *Pachyotoma alpa* are deposited in the Zadock Thompson Natural History Collection at the University of Vermont, Burlington, Vermont, USA.

All collections were made by extracting a plug of sand or soil using a commercial bulb planter. Sand/soil plugs were placed in plastic bags in the field and transported to the laboratory. In the laboratory each plug was washed in water, the water was filtered using commercial coffee maker filters, and the content of the filters was extracted in Berlese funnels (with 15 watts light bulbs) for three days. All sand plugs were moist, as completely dry sand cannot be extracted with a bulb planter. Most samples included only sand, but others contain variable amounts of surface plant debris and the species collected could be either in the sand or on the surface plant debris. The authors collected all samples as follows (localities I and O did not contain examples of the species treated here and are not listed below):

A. Vermont, Chittenden Co., Burlington, Oakledge Park, N44.45640 W73.22513, sand, 24 September 2005.

B. Vermont, Chittenden Co., Burlington, Pine Street Canal, near water treatment plant at south end of Battery St., N 44.46859 W 73.21901, sand, 24 September 2005.

C. Vermont, Chittenden Co., Colchester, Mallets Bay, Colchester Beach, N44.54555 W73.21572, sand with sparse remains of aquatic plant debris, 1 October 2005.

D. Vermont, Chittenden, Colchester, Delta Park, mouth of Winooski River, N44.53111 W73.27396, sand, October 2005

E. Vermont, Chittenden Co., Colchester, Delta Park, beach, intersection of Widemere Way and Horizon View St., N44.53647 W73.27739, sand, October 2005.

F. Vermont, Chittenden Co., Milton, Sand Bar State Park, N44.62818 W73.23769, sand, October 2005.

G. Vermont, Grand Isle Co, South Hero, White’s Beach, N44.62189 W73.32273, sand and thick layer of aquatic plant debris, October 2005

H. Vermont, Grand Isle Co., Grand Isle, Pearl Bay, west of intersection of East Shore North Rd. and Hide Point West Rd., N44.73078 W73.26401, sand with sparse remains of aquatic plant debris, October 2005

J. Vermont, Grand Isle Co., North Hero, Knight Point State Park, eastern beach, N44.76986 W73.29399, sand with sparse remains of aquatic plant debris, October 2005.

K. Vermont, Grand Isle Co., Alburg, Alburg Dunes State Park, N44.86536 W73.30001, sand, October 2005

L. Vermont, Franklin Co., St. Albans, St. Albans Bay State Park, N44.80997 W73.14508, sand with sparse remains of aquatic plant debris, October 2005.

M. New York, Essex Co., Crown Point Historic Area, beach near ruins of Ft. Saint Frederic, N 44.03093 W 73.42768, sand and thick layer of aquatic plant debris, 9 September 2006.

N. Vermont, Addison Co., Chimney Point State Park, N44.03437 W73.42073, sand and thick layer of aquatic plant debris, 9 September 2006.

P. New York, Clinton Co., Ausable Marsh State Wildlife Management Area, N44.57269 W73.43242, sand with sparse remains of aquatic plant debris, 10 September 2006.

Q. Vermont, Orleans, Co., Westmore, Lake Willoughby northeast shore, N44.76906 W72.05338, sand with sparse remains of aquatic plant debris, 5 August 2007.

R. Vermont, Orleans, Co., Westmore, Lake Willoughby south shore, N44.71795 W72.02997, sand with sparse remains of aquatic plant debris, 5 August 2007.

S. Vermont, Orleans Co., Norton, Great Averrill Pond northwest shore, N44.99117 W71.72055, sand, 16 October 2008.

T. Vermont, Orleans, Co., Averill, Great Averill Pond, south shore, N44.97303 W71.68575, sand, 16 October 2008.

U. Vermont, Chittenden Co., South Burlington, University of Vermont Constructed Wetland, N 44.45869 W 73.18936, June 2005.

## Descriptions

### Genus *Ballistura* Börner, 1906

#### Ballistura rossi

Soto-Adames & Giordano sp. n.

urn:lsid:zoobank.org:act:AE48D35A-87D9-4D8C-B69A-B3B8CF8207BB

http://species-id.net/wiki/Ballistura_rossi

##### Material Examined.

Holotype– Female, locality U, slide mounted. Paratypes– locality U, 15 on slides, 3 in alcohol.

##### Type Locality.

USA,Vermont, Chittenden Co., South Burlington, University of Vermont Constructed Wetland, N 44.45869 W 73.18936.

##### Etymology.

The new species is dedicated to Ross Bell in celebration of his contributions to our understanding of the entomological fauna of Vermont.

##### Description.

**Figures 1–13.**
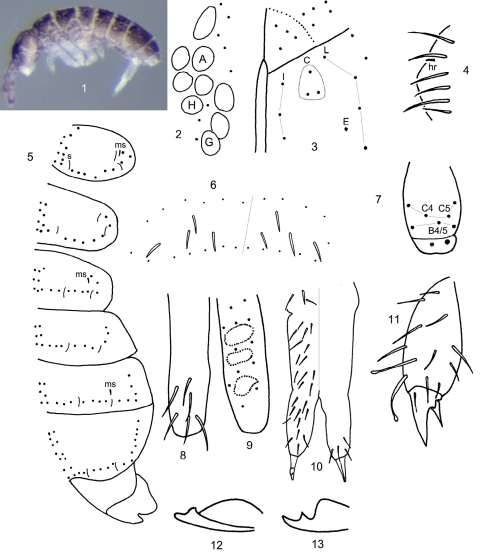
*Ballistura rossi* sp. n. **1** Habitus **2** Eye patch and PAO **3** Labial and postlabial chaetotaxy **4** Lateral anal valve chaetotaxy **5** Chaetotaxy of Th. 2-Abd. 4 **6** Chaetotaxy of Abd. 5 **7** Posterior chaetotaxy of mesotibiotarsus **8** ventral chaetotaxy of dens **9** Dorsal chaetotaxy of dens, tubercles represented by dotted line **10** General anterior (left side) and posterior (right side) chaetotaxy of furcula **11** Metatibiotarsus and claw complex **12–13** Mucro from two individuals.

Length to 0.5 mm. Live individuals black, alcohol preserved specimens (Fig. 1) purple, with pigment more or less uniformly distributed throughout head, body and antennae, legs and manubrium purplish brown. Ant. 4 without basal microsensilla, with 8-9 well developed thin-walled sensilla, and 2-3 additional poorly developed sensilla distributed along distal 2/3 of segment; subapical sense organ with 1 differentiated microsensilla and 1 microrod in a pit. Ant. 3 with 0-1 basal microsensilla; sense organ with 2 clubbed sensilla and 2 differentiated guard sensilla; 1 lateral sensilla present. Ant. 2 with 2 basal microsensilla and 1 distal sensilla. Ant. 1 with 2 basal microsensilla, and 1 whorl of hairs comprising 11 acuminate setae and 2 sensilla. Eyes 8+8, H slightly smaller or subequal to C (Fig. 2), with 3 interocellar setae; PAO circular to elliptical, about 1-1.7X diameter of eye B, and 3 associated setae. Prelabral and labral chaetotaxy 2/554; distal labral margin smooth. Papilla of outer maxillary lobe bifurcate, sublobal plate with 2 appendages. Maxilla with lamella 1 narrow, surpassing tip of capitulum and ciliate only along external margins. Labial palps with a full complement of papillae and 3 proximal setae; papillae E with blunt lateral process and 6 guard setae, e7 absent; labial triangle with 5 anterior and 4 posterior setae; distribution of postlabial setae in columns I, C, E and L as 3,3,1,3/4 (Fig. 3). Body dorsally covered by smooth hairs; some hairs on the pre-posterior row reaching base of setae on posterior row; tergal macrochaetae undifferentiated; thorax without ventral setae. Axial setae on Th. 2-Abd. 3 as 5-6,4//3,3,3; Th. 3 with 14-16 setae on posterior row; microsensillar and sensillar formulae 10//101 and 33//22224, respectively (Figs. 5-6); antero-lateral sensilla on Th. 2 anterior to microsensilla, lateral sensilla on Th. 2-3 anterior to medial sensilla; medial thoracic sensilla inserted on preposterior row of setae, abdominal sensilla inserted just anterior or on posterior row of setae; lateral sensilla on Abd. 5 similar to medial sensilla (Fig. 6). Proximal and medial subcoxae on legs 1-3 with 1,1; 1,5; 3,5-6 setae. Lateral valve of Abd. 6 with 1 hr seta (Fig. 4). Tibiotarsi on legs 1-3 with 20, 20, 22 setae, respectively; tibiotarsal whorl B with B4/5 (Fig. 7); only male seen apparently in reproductive quiescent instar, without modified metatibiotarsal setae; legs 1-3 with 1, 2, 2 clearly capitate tenent hairs (Fig. 11). Unguis and unguiculus toothless, unguiculus lanceolate or acuminate. Ventral tube with 4+4 apical and 1+1 posterior setae. Tenaculum with 3+3 teeth and 1 seta. Anterior and posterior furcal subcoxae with 8-11 and 4-5 setae, respectively. Proportion manubrium/dens/mucro as 3/2/1. Manubrium with 13 dorsal and 0 ventral setae (Fig. 10). Dens weakly tuberculate, with 11 (12) dorsal (Fig. 9-10) and 5 (4/6) ventral setae (Fig. 8, 10). Mucro bidentate, with a pronounced basal membrane (Figs. 12-13).

##### Remarks.

Following Potapov (2001), *Ballistura rossi* sp. n. is most similar to *Ballistura hankoi* (Stach), 1929 from which it can be distinguished (Table 1) by the number of dorsal manubrial setae (13 in *rossei*, 20 in *hankoi*), number of setae around the PAO (3 in *rossei*, 2 in *hankoi*), and number of dorsal setae on dens (11-12 in *rossi*, 10 in *hankoi*). *Ballistura tuberculata* (Stach), 1947 (if this is really different from *Ballistura hankoi*) can be separated from *Ballistura rossi* by the same characters of the furcula and PAO as *Ballistura hankoi*, and by coloration (pale blue-grey in *tuberculata*, dark purple in *rossi*), size (largest individual of *rossi* is 0.5 mm whereas *tuberculata* reaches 0.9 mm) and, possibly, shape of the basal membrane of the mucro (wider at middle in *rossi*, wider on basal half in *tuberculata*). Other chaetotaxic characters may distinguish these three species, but practically nothing has been published about the chaetotaxy *Ballistura hankoi* or *Ballistura tuberculata* (Potapov 2001).

Christiansen and Bellinger (1998) reported *Ballistura tuberculata* from Indiana and Nova Scotia, but whereas the relatively large size of those individuals (up to 0.8 mm) support the determinationas *tuberculata*, the relative size of the OPA and shape of the mucronal membrane suggest similarities to *hankoi*. The specimens from Vermont fit the general description provided by Christiansen and Bellinger (1998) for *Ballistura tuberculata*, except for the larger number of dorsal setae on the dens and the smaller size of the Vermont specimens (Table 1).

*Ballistura rossi* sp. n. appears to be unique among *Ballistura* sp. in having 2 instead of 3 appendages in the sublobal plate of the outer maxillary lobe. However, this character has been reported in relatively few of the species currently placed in *Ballistura* and further information is needed to determine how unique the condition in *Ballistura rossi* sp. n. is.

**Table 1.** Comparison between *Ballistura rossi* sp. n., *Ballistura hankoi* and *Ballistura tuberculata*. Characters for *Ballistura hankoi* and *Ballistura tuberculata* from Europe according to Stach (1947) and Potapov (2001). Characters for *Ballistura tuberculata* from Indiana/Nova Scotia follow Christiansen and Bellinger (1998).

Species Character	*Ballistura rossi* sp. n.	*Ballistura hankoi* Europe (Stach)	*Ballistura tuberculata* Europe (Stach)	*Ballistura tuberculata* Indiana/Nova Scotia (Stach)
Color	deep purple	dark blue	pale bluish grey	blue
PAO/Nearest Eye	1-1.7	≈1	1.5	1.1-1.2
Largest Specimen(mm)	0.5	0.5	0.9	0.8
Dorsal Setae Manubrium	13	20	20	?
Mucro:Dens	1:2	1:2	1:3	1:3
Dorsal Setae on Dens	11-12	10	10	9-10
Mucronal Membrane	wider at middle	wider at middle	wider on basal half	wider at middle

### Genus *Subisotoma* Stach, 1947

#### Subisotoma joycei

Soto-Adames & Giordano sp. n.

urn:lsid:zoobank.org:act:12F3C3E4-5E9E-4345-9DE6-E9D884E34410

http://species-id.net/wiki/Subisotoma_joycei

##### Material Examined.

Holotype– Locality G, Male, slide mounted. Paratypes– Locality G, 3 on slides, 3 in alcohol; H, 2 on slides, 2 in alcohol. Other Material– Locality F (9 in alcohol),

##### Type Locality.

USA,Vermont, Grand Isle Co., South Hero, White’s Beach, N44.62189 W73.32273.

##### Etymology.

This species is dedicated to Joyce Bell for her contributions and support to the study of the arthropod fauna of Vermont.

##### Description.

**Figures 14–25.**
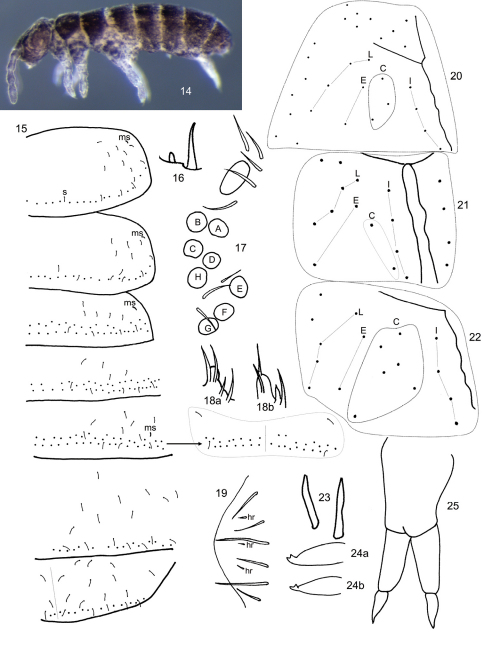
*Subisotoma joycei* sp. n. **14** Habitus of holotype **15** Sensillar chaetotaxy of Th. 2-Abd. 5, s=sensilla, ms= microsensilla, arrow points at position of Abd. 3 medial sensilla in a different individual **16** Subapical organ of Ant. 4 **17** Eye patch and PAO **18** Labial palp papilla E **18a** dorsal aspect **18b** ventral aspect **19** Lateral anal valve **20–22** Postlabial chaetotaxy showing variation in number of setae assigned to column C **23** Modified metatibiotarsal seta in mature male **24** Mucro **24a** lateral aspect **24b** oblique aspect **25** Organization of furcula.

**Figures 26–28.**
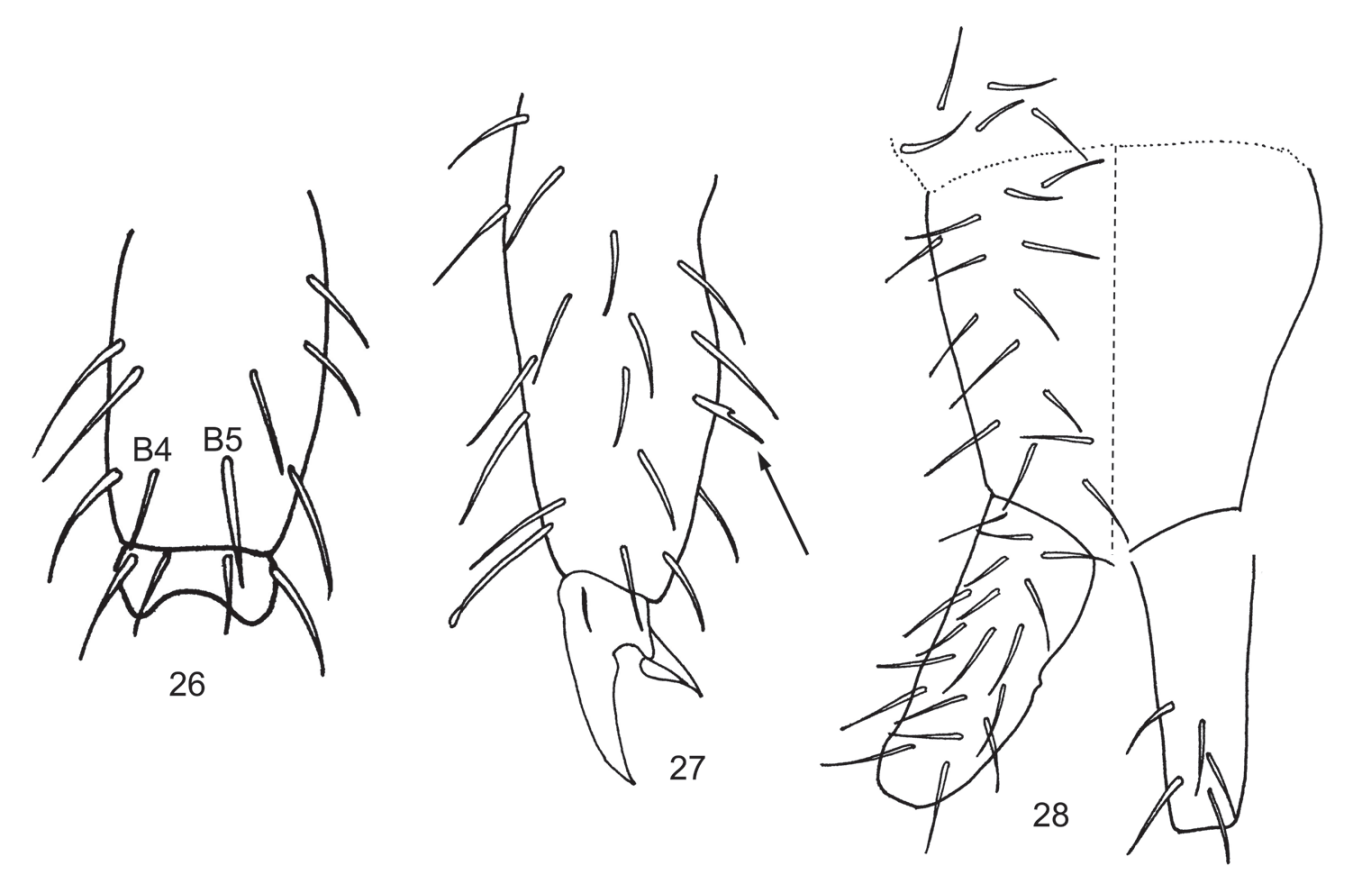
*Subisotoma joyce*i sp. n. **26** Posterior view of mesothoracic tibiotarsus **27** Metatibiotarsus, arrow points at abnormal seta **28** Dorsal (left side) and ventral (right side) chaetotaxy of manubrial base, manubrium and dens.

Length to 1.2 mm. Living individuals black, alcohol preserved specimens dark purplish brown to black, with pigment more or less uniformly distributed through out head, body and antennae, legs and manubrium purplish (Fig. 14); some individuals with paired black longitudinal lines extending from Th. 2-Abd. 3. General body shape short and stout, with sudden bend between Abd. 4-5 (Fig. 14) as in *Folsomides*. Ant. 4 without basal microsensilla (bms of Potapov 2001), with at least 13 well-developed thin-walled sensilla, and 14 other sensilla distributed along the length of the segment; subapical sense organ with 1 differentiated microsensilla and 1 microrod in a pit (Fig. 16). Ant. 3 with 1 poorly differentiated basal microsensilla; sense organ with 2 clubbed sensilla and 2 differentiated guard sensilla; 1 lateral sensilla present; females with 9-10, and males with up to 16 additional sensilla distributed mostly on dorsal face of segment. Ant. 2 with 3 basal microsensilla; and 1-2 distal sensilla. Ant. 1 with 2 basal microsensilla, 17-18 smooth, acuminate setae, and 2-3 sensilla. Eye patch with 8+8 subequal eyes and 3 interocellar setae; PAO elliptical, about 2X diameter of eye B, and 4-5 associated setae (Fig. 17). Prelabral and labral chaetotaxy 2/554; distal labral margin smooth. Papilla of outer maxillary lobe simple, sublobal plate with 4 appendages. Maxilla with lamella 1 narrow, with cilia only along margins, surpassing tip of capitulum. Labial palps with a full complement of papillae and 3 proximal setae; papillae E with lateral process and 6 guard setae, seta e7 absent (Fig. 18a-b); labial triangle with 5 anterior and 4 posterior setae; distribution of postlabial setae in columns I, C, E and L variable 4(5-6), 3/5 (1,6), 2-3, 5(4) (Figs. 20-22). Body dorsally covered by smooth hairs; some hairs on the pre-posterior row reaching base of setae on posterior row; tergal macrochaetae undifferentiated; thorax without ventral setae. Axial setae on Th. 2-Abd. 3 as 7-9; 7-8(9)//5(4/6-7); 5(3-4/6); 5. Th. 3 with 28/33(20) setae on posterior row; microsensillar formula 10(1)//101; sensillar formula variable, often asymmetric 14(17-18); 9-13//9(8/10); 8(9); 9(5/8/10); 11-19; 12(4/8/14/16) (Fig. 15); antero-lateral sensilla on Th. 2 posterior to microsensilla, but spatial relation among lateral sensilla variable between individuals; lateral sensilla on Th. 2-3 anterior to medial sensilla; medial thoracic sensilla inserted on preposterior row of setae, insertion of abdominal sensilla variable, just anterior or on posterior row of setae, or clearly anterior to subposterior row (Fig. 15); all sensilla on Abd. 5 similar in size. Proximal and medial subcoxae on legs 1-3 with 1, 0; 5-8, 4-9; 6-12, 6-15 setae. Lateral valve of Abd. 6 with 3 hr setae (Fig. 19). Ventral thoracic setae absent. Sculpturing of thoracic sterna smooth. Tibiotarsi 1-3 with 24, 27, 30 setae; tibiotarsal whorl B complete, with B5 clearly thicker and longer than B4 (Fig. 26); adult males with setae B5 and x truncate (Fig. 23); legs 1-3 with 1 (A1), 2 (A1, A7), 2 (A1, B7) capitate or acuminate tenent hairs (Fig. 27). Unguis and unguiculus toothless, unguiculus triangular. Ventral tube with 6-11 disto-lateral and 6-9 posterior setae. Tenaculum with 3+3 teeth and 1 seta. Sterna of Abd. 3 without isolated field of setae. Anterior and posterior furcal subcoxae with 9-19 and 4-11 setae, respectively. Proportion manubrium/dens/mucro as 3/2/1. Dens smooth, and cylindrical (Fig. 25). Chaetotaxy of manubrium and dens as in Fig. 28: manubrium with 5-8 basal setae, 15-24 dorsal and 0 ventral setae; dens with 18-20 (15/26) dorsal and 5 (4/6) ventral setae. Mucro with wide lamella, without basal notches, clearly separated from dens; bidentate, teeth subequal (Fig. 24a-b).

##### Remarks.

Three individuals show variation in the number and size of eyes. In the small juvenile eyes G and H are small, barely rising above the cuticle; one male is blind; and in one female all eyes in one patch are subequal, but on the other patch eye E is less than half the size of F. The axial setae are often disorganized and the number of setae in a column is open to interpretation. The number of tergal sensilla is variable. Most individuals have an asymmetric number of sensilla, and two individuals lack the microsensilla of Abd. 1 on one side. Tenent hair B7 on metathoracic legs often appears acuminate instead of capitate. Two individuals have 3+4 tenacular teeth.

The generic placement of the new species is problematic. It better fits in the genus *Subisotoma* (Table 2), but it is unique among species currently assigned to that genus in having more than 8+8 tergal sensilla on each segment, by the significantly larger number of dental setae (most *Subisotoma* species have 4 or fewer dorsal and 1-2 ventral setae, whereas *Subisotoma joycei* has 18-20 dorsal and 4-6 ventral setae), and by having a well developed furcula with mucro exhibiting a wide lamella and clearly separated from the dens. *Subisotoma joycei* is similar to species in the genus *Isotopenola* Potapov, Babenko, Fjellberg & Greenslade, 2009 in the presence of sensillar polychaetosis on body terga, but differs from all forms in that genus by having smooth thoracic sterna, lacking an isolated field of setae on Abd. 3 sterna and in the number of guard setae on labial papilla E. The strong polychaetotic furcula in *Subisotoma joycei* resembles the condition in *Ballistura*, but the new species clearly differs from *Ballistura* in maxillary palp structure, sensillar and microsensillar formulae, presence of a full complement of setae in tibiotarsal whorl B, and dens sculpturing (Table 2).

The new species is most similar to *Ballistura ewingi* James, 1933, *sensu* Folsom (1937) from which it differs in aspects of color pattern (trunk ventrally white in *ewingi*, dark purple brown in *joycei*), the number of tenent hairs (2-3 on all legs in *ewingi*, 1, 2, 2 in *joycei*), number of distal seta on ventral tube (4 on *ewingi*, 11 in *joycei*), and number of tenacular teeth (2 in *ewingi*, 3-4 in *joycei*). The new species may be the same as the Pennsylvania specimens preliminarily assigned to *Ballistura ewingi* by Christiansen and Bellinger (1998), although this form also seems to have considerably fewer distal setae on the ventral tube than S. *joycei* sp. n.(Table 3). *Ballistura ewingi* has been described as having smooth dens, and probably does not belong in *Ballistura*, which Potapov (2001) restricts to species with tuberculate dens. Important characters needed to determine the appropriate generic placement of *Ballistura ewingi* remain undescribed and require the study of fresh material.

The new species is also similar to *Ballistura excavata* Folsom, 1937, but the two species are easily separated by body color, eye number, shape of tenent hairs and unguiculus, and structure of the dens (Table 3).

**Table 2.** Diagnostic characters for selected genera in the *Proisotoma* genera complex in comparison with *Subisotoma joycei* sp. n. All genera, retained in the sense of Potapov (2001) or Potapov et al. (2006, 2009).

Genus Character	*Scutisotoma*	*Proisotoma*	*Folsomides*	*Ballistura*	*Isotopenola*	*Subisotoma*	*Subisotoma joycei* sp. n.
Prelabral Seta	4	3	2	2	2	2	2
Outer Maxillary Palp/Sublobular Appendages	bifurcate/4	simple/4	simplebifurcate/3	bifurcate/3	simple/4	simple/4	simple/4
Number Guard Setae on Labial Papilla E	7	5– e4 and e7 absent	7	6– e7 absent	4-5	6– e7 absent	6– e7 absent
Tergal Microsensillar Formula	11//111	10//00000//000	10//000 to11//111	11//111	10//10111//111	10//000, 10//100, 10//001, 10//101	10//101
Position Medial Sensilla Abd. 3	medial row	posterior row	medial row	just anterior to posterior row	subposterior row	posterior row or just anterior to posterior row	posterior row or just anterior to posterior row
Sternal Thoracic Setae	presentabsent	presentabsent	absent	absent	absent	absentpresent	absent
Mesotibiotarsal Setae B4/5	absent	absent	present	present	absent	absent	absent
Ventral Manubrial Setae	10	1	absent	0	0	0	0
Dorsal/Ventral Dental Setae	variable	3-7/4-6	2-6/0-3	variable	3-8/1	<4/1	18-20/4-6
Tergal Sensilla Polychaetosis	absent present	absent	absent	absent	absent present	absent	present
Dental Sculpturing	crenulatetuberculategranulate	crenulate	smooth	tuberculate	smooth	smooth	smooth

**Table 3.** Comparison between *Subisotoma joycei* sp. n., *Ballistura excavata* andtwo forms of *‘Ballistura’ ewingi*. The presence of smooth dens places *Ballistura ewingi* outside of *Ballistura* as currently diagnosed by Potapov (2001), but the species is retained in that genus pending study of fresh material.

Species Character	*Subisotoma joycei* sp. n.	*‘Ballistura’ ewingi* James Mississippi	*‘Ballistura’ ‘ewingi’* Christiansen & Belliger Pennsylvania	*Ballistura excavata* Folsom
Eye Number	8	8	8	6
Color	dark purple	dark purple	dark purple	yellow
Tenent Hairs on L1-3	1,2,2all capitate	2-3 on all legsall capitate	1,2,2all capitate	?acuminate
Unguiculus Shape	triangular, asymmetric	triangular, asymmetric	triangular, asymmetric	triangular, symmetric
Unguiculus Apical Filament	absent	absent	absent	present
Ventral Tube Distal Setae	11	4	4	?
Teeth on Tenaculum	3-4	2	3	4
Dorsal Cuticle of Dens	smooth	smooth	smooth	tuberculate
Dorsal Seta on Dens	18-20	18? (Folsom 1937, Fig. 128)	?	14-16? (Folsom 1937, Fig. 136)
Ventral Setae on Dens	4-6	6	5	at least 2(Folsom 1937, Fig. 136)

### Genus *Scutisotoma* Bagnall, 1949

#### Scutisotoma champi

Soto-Adames & Giordano sp. n.

urn:lsid:zoobank.org:act:0EA01C0B-4A76-4DEE-B863-26F4952B5013

http://species-id.net/wiki/Scutisotoma_champi

##### Material.

Holotype– Female, slide mounted, locality B. Paratypes– Locality B, 9 individuals on slides, 1976 in alcohol; N, 5 mounted on two slides and more than 3000 in alcohol; P, 4 individuals on slides and 59 in alcohol; Other material– Locality A, 800 individuals in alcohol; C, 7 in alcohol; D, 187 in alcohol; E, 30 in alcohol; F, 32 in alcohol; G 1186 in alcohol; H, 1290 in alcohol; J, 158 in alcohol; K, 35 in alcohol; L, 1723 in alcohol; M, 1555 in alcohol; Q, 5 in alcohol; R, 2 in alcohol; S, 1 in alcohol; T, 8 in alcohol.

##### Type Locality.

USA, Vermont, Chittenden Co., Burlington, Pine Street Canal, near water treatment plant at south end of Battery St., N 44.46859 W 73.21901

##### Etymology.

The species is named after ‘Champ’ the denizen monster of Lake Champlain, were the new species seems to be most abundant.

##### Description.

**Figures 29–40.**
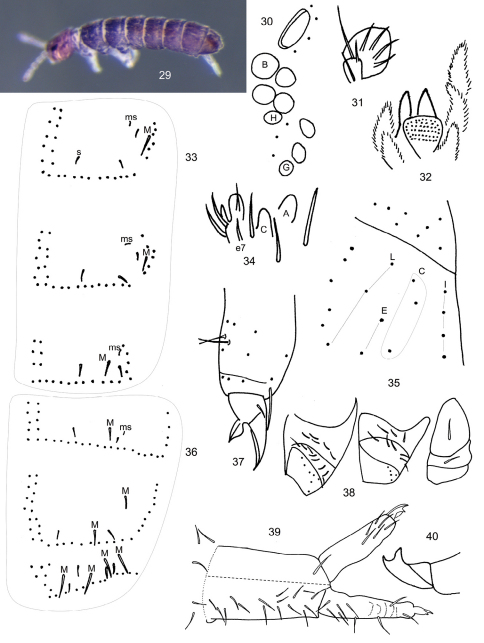
*Scutisotoma champi* sp. n. **29** Habitus **30** Eye patch and PAO **31** Outer maxillary lobe **32** Frontal aspect of maxilla **33** Dorsal chaetotaxy of Th. 2-Abd. 1, s= sensilla, ms= microsensilla, M= macroseta **34** Ventral aspect of labial palp, terminal process of papilla A, C and E omitted **35** Labial and postlabial chaetotaxy **36** Dorsal chaetotaxy of Abd. 3-Abd. 5 **37** Male metathoracic leg, lateral view **38** Chaetotaxy of pro-, meso- and meta thoracic subcoxae, from left to right, respectively **39** Dorsal (lower half) and ventral (upper half) chaetotaxy of furcula **40** mucro.

Length to 0.7 mm. Live specimens black, alcohol preserved individuals (Fig. 29) dark purple, with pigment more or less uniformly distributed throughout head, body and antennae; legs and manubrium light purplish brown. Ant. 4 with 1 basal microsensilla, and 7 poorly differentiated thin-walled sensilla distributed along apical half of segment; subapical sense organ with 1 poorly differentiated microsensilla and 1 micropeg in a pit. Ant. 3 with 1 basal microsensilla; sense organ with 2 clubbed sensilla and 2 differentiated guard sensilla; 1 lateral sensilla present; males with 2 additional dorsal sensilla. Ant. 2 with 3 basal microsensilla and 1 distal sensilla. Ant. 1 with 2 basal microsensilla and 1 whorl comprising 11 setae and 2 sensilla. Eyes 8+8, G and H slightly smaller than others (Fig. 30), with 3 interocellar setae; PAO elliptical, about 2.3X diameter of eye B, with 4-5 associated setae. Prelabral and labral chaetotaxy 4/554; distal labral margin smooth. Papilla of outer maxillary lobe bifurcate, sublobal plate with 4 appendages (Fig. 31). Maxilla with lamella 1 narrow, surpassing the tip of capitulum, with cilia confined to external margins (Fig. 32). Labial palps with a full complement of papillae and 3 proximal setae; papillae E with blunt lateral process and 7 guards, e7 detached from papilla (Fig. 34); labial triangle with 5 anterior and 4 posterior setae; distribution of postlabial setae in columns I, C, E and L as 4,3,2,3 (Fig. 35). Body dorsally covered by smooth hairs; some hairs on the pre-posterior row reaching base of setae on posterior row; thorax without ventral setae; axial setae on Th. 2-Abd. 3 as 6-8, 6-7//4-5, 4-5, 4-5; Th. 3 with 20-22 setae on posterior row; microsensillar and sensillar formulae 11//111 and 33//22224, respectively (Figs. 33, 36); lateral sensilla on Abd. 5 swollen (Fig. 36), all sensilla inserted anterior to posterior row of setae, tergal macrochaetae smooth, poorly differentiated, distributed as 11//11124; Abd. 5≈ 3.2X medial macrochaeta. Proximal and medial subcoxae on legs 1-3 with 1, 1; 3(4), 6(7); 5(4,6,7), 7(6,8) setae (Fig. 38). Lateral valve of Abd. 6 similar to *Ballistura joycei*, with 3 hr setae. Tibiotarsal whorl B complete; male metatibiotarsal setae x and B5 thin, short, bothriotrica-like, with modified sockets (Fig. 37); legs 1-3 with 1,2,2 tenent hairs as in *Ballistura joycei*, but all tenent hairs acuminate, A1 on L2 sometimes appearing weakly clubbed. Unguis and unguiculus toothless (Fig. 37). Ventral tube with 3+3 apical setae, posterior face with 1 basal and 4 distal setae. Tenaculum with 4+4 teeth and 1 seta. Anterior and posterior furcal subcoxae with 12-18 and 7-8 setae, respectively. Proportion manubrium/dens/mucro as 6/4/1. Chaetotaxy of furcula as in Figure 39: manubrium with 17-18 dorsal and 1 ventral setae; dens weakly crenulated, with 9 dorsal and 6 ventral setae. Mucro bidentate, subapical tooth longer than apical (Fig. 40).

##### Remarks.

*Scutisotoma champi* sp. n. is unique among *Scutisotoma* species in having a lamelate bidentate mucro, 4 tenacular teeth, 9 dorsal and 6 ventral setae on dens, and maxillar lamela 1 longer then the capitulum. The new species is most similar to the Central Asian *Scutisotoma acorrelata* Potapov, Babenko & Fjellberg, 2006 and *Scutisotoma tenuidentifera* Potapov, Babenko & Fjellberg, 2006, from which it can be distinguished by the characters listed in Table 4. Among North American species, *Scutisotoma champi* sp. n. is most similar to *Scutisotoma titusi* (Folsom), 1937 from which it can be easily distinguished by the number of ventral setae on dens and the number of tergal sensilla on Abd. 4 and 5 (2+2, 4+4 in *champi*, 7-8+7-8, 8-12+8-12 in *titusi*).

*Scutisotoma champi* sp. n. is the most common species found in sandy beaches on the northern 2/3 of Lake Champlain as well as Lake Willoughby and Greater Averill Pond, and it is likely present in most if not all lakes and large ponds in northern Vermont and southern Quebec. The species was collected in apparently healthy beaches (e.g., Pearl Bay, locality H), as well as on highly disturbed, strongly compacted beaches (e.g., Colchester Beach, locality C). The species is most abundant in beaches with aquatic plant litter, but it is also found in sand in beaches without visible surface plant remains.

#### Genus Pachyotoma

Bagnall, 1949

http://species-id.net/wiki/Pachyotoma

Pachyotoma alpa (Christiansen & Bellinger), 1980 new combination
Ballistura alpa Christiansen & Bellinger, 1980: 581 (Mt. Washington, New Hampshire, USA)

##### Material Examined.

USA, Vermont, Chittenden Co., Bolton, Camel’s Hump ≈1200-1230 m elevation, 7 August 1972, W. Rittenhouse, coll. Two slides, one with three individuals, the other with one individual. These are the individuals originally determined as *Ballistura alpa* by Bellinger (1982).

**Figures 41–46.**
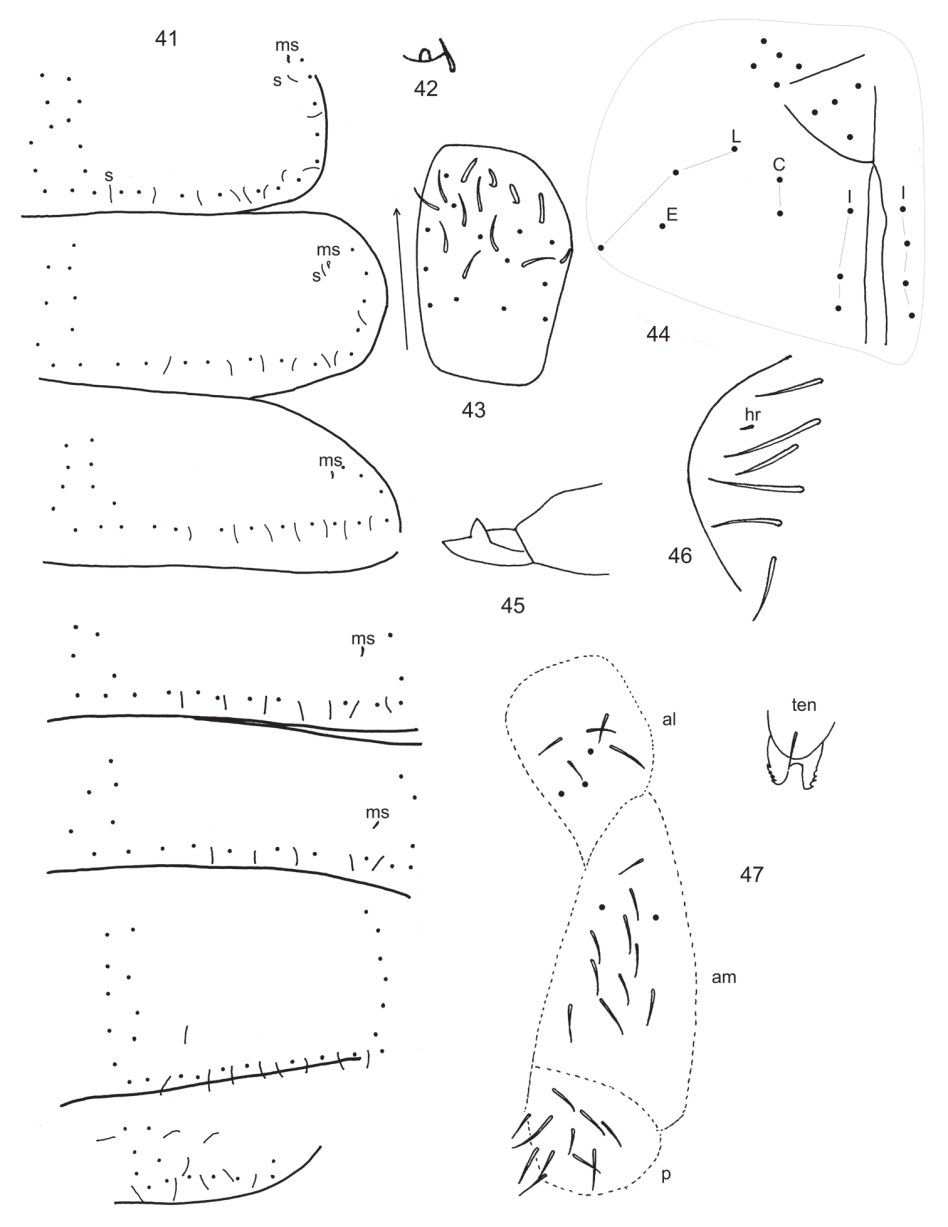
*Pachyotoma alpa* (Christiansen & Bellinger) **41** dorsal chaetotaxy of Th. 2-Abd. 5, s= sensilla, ms= microsensilla **42** Ant. 4 subapical sense organ **43** Ant. 3 sense organ and associated sensilla, arrow points anteriorly **45** Mucro **46** Lateral anal valve **47** Chaetotaxy of anterolateral (al), anteromedial (am) and posterior (p) furcula subcoxae and their position relative to tenaculum (ten).

The following notes serve as a complement to the original description. Cuticle covered by many secondary granules. Organite of Ant. 4 capitate, guard sensilla not strongly modified (Fig. 42). Ant. 3 sense organ, lateral and supplementary sensilla as in Fig. 43. Ant. 2 with 1 distal sensilla and at least 1 basal microsensilla. Ant. 1 with 2 weakly modified basal microsensilla, 3-4 sensilla, and 12-14 setae. Eye patch with 3 setae, PAO with 2 guard setae. Labral formula 4/5,5,4. Apical seta of outer maxillary lobe bifurcate, sublobal plate with 4 appendages. Maxillary lamellae not clearly seen, but lamella 1 apparently surpassing tip of capitulum and apically acuminate (lateral margins converging well before tip of lamella), with cilia only along lateral margins. Labial papilla E with 7 guard setae, e7 detached from papilla as in *Scutisotoma champi*. Labial triangle with 5 anterior and 4 posterior setae. Postlabium with 3(4), 2, 1, 3 setae in columns I, C, E and L, respectively (Fig. 44). Body setae smooth and short, tip of seta on anterior rows not reaching base of setae on posterior rows. Thoracic sterna without hairs. Axial setae on Th. 2-Abd. 3 as 3-4, 5//4, 2-3, 2-4; Th. 3 with 18-20 setae on posterior row; microsensillar and sensillar formulae as 11//111 and 8(10)8//7758(9)7, respectively (Fig. 41); Th. 2 with 2 lateral sensilla inserted anterior to medial sensilla; medial sensilla on all segments inserted on p-row; Abd. 5 sensilla not swollen. Tergal macrochaetae undifferentiated. Proximal and medial subcoxae on legs 1-3 with 1, 0; 4(5), 4; 5(7), 4 setae. Lateral valves of Abd. 6 similar to *Ballistura rossi*, with 1 hr seta (Fig. 46). Tibiotarsal whorl B complete; legs 1-3 with 1, 2, 2 tenent hairs, all acuminate. All unguis with 1 inner tooth; unguiculus toothless, without terminal filament. Ventral tube with 7+7 apical setae, posterior face with 2 basal and 4 distal setae. Tenaculum with 4+4 teeth and 1 seta. Anterolateral, anteromedial and posterior furcal subcoxae with 7/8(6,10), 11 (12,13) and 7-11 setae, respectively (Fig. 47). Manubrium with 15 dorsal and 0 ventral setae. Dens granulate, with 7-8 dorsal and 6 (5) ventral setae. Mucro bidentate, subapical tooth longer than apical (Fig. 45).

##### Remarks.

This species was originally placed in the genus *Ballistura*, but the presence of a complete whorl B on pro- and mesothoracic legs and four prelabral setae excludes it from that genus (Potapov 2001). The presence of secondary granules on the cuticle in combination with a well developed furcula and the insertion of the medial and almost all other tergal sensilla on Th. 2-Abd. 3 on the posterior row of setae place this species in the genus *Pachyotoma*. In the original description of the species Christiansen and Bellinger (1980) indicate the presence of 5 distal setae on the ventral tube and a toothless unguis. The individuals from Vermont have 7 distal setae on the ventral tube and one distinct inner tooth on all unguis and may represent a distinct species.

**Table 4.** Comparison between *Scutisotoma champi* sp. n. and similar species.

Species Character	*Scutisotoma champi* sp. n.	*Scutisotoma acorrelata* Potapov, Babenko & Fjellberg	*Scutisotoma tenuidentifera* Potapov, Babenko & Fjellberg	*Scutisotoma titusi* (Folsom)
Maxillary Lamella 1	longer than capitulum of maxilla	shorter than capitulum of maxilla	shorter than capitulum of maxilla	shorter than capitulum of maxilla
Abd. V Anteriormost Sensilla	s2	s3	s3	s1
Ventral Tube Distal Setae	3	4	4	5-7
Tenacular Teeth	4	3	3	4
Dorsal Setae on Dens	9	10-11	6-7	14-15
Number Mucronal Teeth	2	2	3	3

## Supplementary Material

XML Treatment for Ballistura rossi

XML Treatment for Subisotoma joycei

XML Treatment for Scutisotoma champi

XML Treatment for Pachyotoma

## References

[B1] Bagnall RS (1949) Contributions towards a knowledge of the Isotomidae (Collembola). I-IV. Annals and Magazine of Natural History 1: 529-541

[B2] Bellinger PF (1982) Collembola from Vermont. Entomological News 93: 180-182

[B3] Börner C (1906) Das System der Collembolen nebst Beschreibung neuer Collembolen des Hamburger Naturhistorischen Museums. Mitteilungen aus dem Naturhistorischen Museum 23: 147-188

[B4] Christiansen K Bellinger P (1980) The Collembola of North America north of the Rio Grande, a taxonomic analysis. 1^st^ ed., Grinnell College, Grinnell, Iowa, 1322p.

[B5] Christiansen K Bellinger P (1998) The Collembola of North America north of the Rio Grande; A taxonomic analysis. 2^nd^ ed., Grinnell College, Grinnell, Iowa, 1518 pp.

[B6] Fjellberg A (2007) The Collembola of Fennoscandia and Denmark. Part II: Entomobryomorpha and Symphypleona. Fauna Entomologica Scandinavica 42: 1-264

[B7] Folsom JW (1937) Nearctic Collembola or springtails of the family Isotomidae. United States National Museum Bulletin 168: 1-144 doi: 10.5479/si.03629236.168.1

[B8] James HG (1933) Collembola of the Toronto region with notes on the biology of *Isotoma palustris* Mueller. Transactions of the Royal Canadian Institute. 29: 77–116 + 4 plates.

[B9] Potapov M (2001) Synopses on Palaearctic Collembola, Volume 3, Isotomidae. Abhandlungen und Berichte des Naturkundemuseums, Görlitz 73: 1-603

[B10] Potapov M Babenko A Fjellberg A (2006) Taxonomy of the *Proisotoma* complex. Redefinition of genera and description of new species of *Scutisotoma* and *Webercantha* (Collembola, Isotomidae). Zootaxa 1382: 1-74

[B11] Potapov M Babenko A Fjellberg A Greenslade P (2009) Taxonomy of the *Proisotoma* complex II. A revision of the genus *Subisotoma* and a description of *Isotopenola* gen. nov. (Collembola: Isotomidae). Zootaxa 2314: 1-40

[B12] Soto-Adames FN (2010) Two new species and descriptive notes for five *Pseudosinella* species (Hexapoda: Collembola: Entomobryidae) from West Virginian (USA) caves. Zootaxa 2331: 1-34

[B13] Stach J (1929) Verzeichnis der Apterygogenea Ungarns. Annales Musei Nationalis Hungarici 26: 1-75

[B14] Stach J (1947) The Apterygotan fauna of Poland in relation to the world fauna of this group of insects. Family Isotomidae. Polska Akademia Umiejetnosci, Krakow 1–488.

